# Inhibition of PI3K/AKT and MEK/ERK pathways act synergistically to enhance antiangiogenic effects of EGCG through activation of FOXO transcription factor

**DOI:** 10.1186/1750-2187-3-7

**Published:** 2008-03-20

**Authors:** Sharmila Shankar, Qinghe Chen, Rakesh K Srivastava

**Affiliations:** 1 Department of Biochemistry, University of Texas Health Science Center at Tyler, Tyler, Texas, 75708-3154, USA

## Abstract

**Background:**

We have recently shown that epigallocatechin-3-gallate (EGCG), a polyphenolic compound from green tea, inhibits angiogenesis. However, the molecular mechanisms by which EGCG inhibits angiogenesis have never been investigated. In this study, we examined the interaction of PI3K/AKT and MEK/ERK pathways on the regulation of FOXO transcription factors, which ultimately control the antiangiogenic effects of EGCG.

**Results:**

Inhibition of PI3K/AKT and MEK/ERK pathways interact synergistically to inhibit migration and capillary tube formation of HUVEC cells and further enhanced the antiangiogenic effects of EGCG. Inhibition of AKT and MEK kinases synergistically induced FOXO transcriptional activity, which was further enhanced in the presence of EGCG. Phosphorylation deficient mutants of FOXO induced FOXO transcriptional activity, inhibited HUVEC cell migration and capillary tube formation. Inhibition of FOXO phosphorylation also enhanced antiangiogenic effects of EGCG through transcriptional activation of FOXO.

**Conclusion:**

Inhibition of PI3K/AKT and MEK/ERK pathways act synergistically to regulate antiangiogenic effects of EGCG through activation of FOXO transcription factors. The activation of FOXO transcription factors through inhibition of these two pathways may have physiological significance in management of diabetic retinopathy, rheumatoid arthritis, psoriasis, cardiovascular diseases, and cancer.

## Background

Angiogenesis is a complex event which requires endothelial cell sprouting, lumen formation, tubulogenesis and is regulated by the coordinated action of different transcription factors [1,2]. Their interaction leads to endothelial cell differentiation and acquisition of arterial, venous and lymphatic properties. The transcription factors FOXO plays a major role in these events [3]. It participates in both embryonic and adult angiogenesis. The FOXO subgroup regulates the correct organization of the vascular system, controlling excessive endothelial growth and inducing apoptosis in both embryos and adult mice. Many members of this family are expressed very early in the developing vasculature. FOXO transcription factors play a crucial role in the regulation of tissue homeostasis in organs such as the pancreas and the ovaries and complex diseases such as diabetes and cancer [4-8]. Despite redundant functions of FOXO proteins in vitro, their in vivo roles in development and physiology are diverse, and genetic loss of the distinct *FOXO *isoforms results in different phenotypes. For example, mice homozygous for a *FOXO1*^-/- ^allele, but not *FOXO3a*^-/- ^or *FOXO4*^-/- ^mice, die during embryogenesis from defects in vascular development [9,10]. Although these studies suggest an essential role of FOXO1 in the formation and maturation of the nascent vasculature, relatively little is known about the function and significance of the distinct Foxo family members for the angiogenic activity of endothelial cells and postnatal vessel formation. In mature endothelial cells, inhibition of FOXO1 activity has been shown to be an important mechanism through which *angiopoietin 1 *(*Ang1*) modulates endothelial function [3].

FOXO subfamily of forkhead transcription factors include FOXO1a/FKHR, FOXO3a/FKHRL1, and FOXO4/AFX [11-14]. The PI3 kinase (PI3K) pathway, via activation of its downstream kinase AKT, phosphorylates each of the FOXO proteins [15-17]. These phosphorylations result in impairment of DNA binding ability and increased binding affinity for the 14-3-3 protein [16,17]. Newly formed 14-3-3-FOXO complexes are then exported from the nucleus [18], thereby inhibiting FOXO-dependent transcription. Inhibition of the PI3K pathway leads to dephosphorylation and nuclear translocation of active FKHRL1, FKHR, and AFX; which induce cells cycle arrest and apoptosis [19]. Conversely, loss of PTEN activity results in increased AKT activity leading to inhibition of FOXO protein activity through phosphorylation and cytoplasmic sequestration. In addition, the data demonstrate that FOXO transcriptional activity controls cellular proliferation and apoptosis downstream of PTEN [20,21]. FOXO regulates cell cycle and apoptotic genes such as cyclin-dependent kinase inhibitor (CKI) p27^/KIP1 ^[18,20,22,23], Bim [24,25], Fas ligand [16], and Bcl-6 [26]. Consequently, activation of the PI3K pathway serves to repress FOXO-mediated growth arrest and apoptosis. However, regulation of FOXO target genes is multifactorial, and therefore other transcription factors and post-translation regulatory events will influence the final level of protein expression. Interestingly, overexpression of AKT, and inactivation and loss of PTEN are frequently observed in pancreatic cancer [27-33], indicating a potential role for FOXOs in modulating both cell cycle and apoptosis during tumorigenesis and treatment. Together, these results indicate that FOXO proteins are important downstream effectors of PTEN tumor suppressive activity; however, their molecular targets and mechanisms of action in regulating angiogenesis are not well understood.

FOXO transcription factors play a crucial role in the regulation of tissue homeostasis in organs such as the pancreas and the ovaries and complex diseases such as diabetes and cancer [4-8]. FOXO transcription factors are emerging as critical transcriptional integrators among pathways regulating differentiation, proliferation, survival, and angiogenesis [10,34-36]. Foxo transcription factors regulate angiogenesis and postnatal neovascularization by regulation angiopoietin 2 (Ang2) and eNOS [36]. Gene expression profiling showed that FOXO1 and FOXO3a specifically regulate a nonredundant but overlapping set of angiogenesis- and vascular remodeling-related genes [36]. The FOXO1-deficient mice died around embryonic day 11 because of defects in the branchial arches and remarkably impaired vascular development of embryos and yolk sacs [10]. Therefore, it is possible that EGCG inhibits angiogenesis by regulating FOXO transcription factors.

The Ras proteins are small (21 kDa) GTP-binding, membrane-associated proteins [37]. They are in their activated state when bound to GTP, and are inactivated by GTP hydrolysis. This intrinsic GTPase activity is enhanced by association with GTPase-activating protein [37]. The Ras proteins transduce signals from ligand-activated tyrosine kinase receptors to downstream effectors [38]. Activating mutations can impair GTP hydrolysis and lead to constitutively activated Ras that impacts the cellular phenotype [39]. Oncogenic Ras can lead to cellular transformation [40], presumably by perturbing its signal transduction pathways. Ras regulates multiple signaling pathways [41]. Three major groups of MAP kinases are found in mammalian cells: extracellular signal-regulated protein kinase (ERK) [42], p38 MAP kinase [43], and c-Jun N-terminal kinase (JNK) [44-46]. MAP kinases regulate many cellular activities, which range from gene expression to mitosis, movement, metabolism, angiogenesis and apoptosis. These MAP kinases are activated by the dual phosphorylations of neighboring threonine and tyrosine residues in response to various extracellular stimuli [47,48]. Specifically, p38 and JNK have been implicated in stress-responsive signaling leading to the initiation of adaptive events such as gene expression, differentiation, metabolism, and apoptosis [44,45,49]. ERKs are often activated by growth signals, such as epidermal growth factor (EGF) or platelet-derived growth factor [50]. FOXO1 (FKHR) contains 15 consensus phosphorylation sites for the MAPK family. Therefore, MAPKs could directly regulate the transcriptional activity of FOXO1 via phosphorylation. *In vitro *kinase assay showed that FOXO1 was phosphorylated by ERK and p38 but not by JNK [51]. In NIH3T3 cells, epidermal growth factor or anisomycin increased phosphorylation of exogenous FOXO1, which was significantly inhibited by pretreatment with an MEK 1 inhibitor, PD98059, or a p38 inhibitor, SB203580. Two-dimensional phosphopeptide mapping using mutation of phosphorylation sites for MAPK revealed that the nine serine residues in FOXO1 are specifically phosphorylated by ERK and that five of the nine residues are phosphorylated by p38 *in vivo*.

Green tea is a popular beverage consumed in some parts of the world and is a rich source of polyphenols [52,53]. Green tea, which is widely consumed in China, Japan and India, contains polyphenolic compounds, which account for 30% of the dry weight of the leaves. A polyphenolic constituent, (-)-epigallocatechin-3-gallate (EGCG), is the major and most effective chemopreventive agent in green tea. Epidemiological studies revealed that the incidences of stomach and prostate cancers are the lowest in the world among a population that consumes green tea on a regular basis [52,54-56]. It acts as an antioxidant, antiproliferative, antitumor, and anti-angiogenic, and thus a novel candidate for chemoprevention [52,55,56]. We showed that EGCG inhibited angiogenesis through Ras/MEK/ERK pathway. However, there are no studies to demonstrate how the inhibition of PI3K/AKT and MEK/ERK pathways interact together to regulate FOXO-dependent angiogenesis.

The purpose of this study was to examine whether the inhibition of PI3K/AKT and MEK/ERK pathways enhance the antiangiogenic effects of EGCG through activation of FOXO transcription factors. The data demonstrate that inhibition of PI3K/AKT and MEK/ERK pathways act synergistically to induce FOXO transcription activity, capillary tube formation and migration. Furthermore, antiangiogenic effects of EGCG are regulated through activation of FOXO transcription factors.

## Results

### Involvement of PI3K/AKT and MEK/ERK pathways on EGCG-induced apoptosis

**Figure 1:**
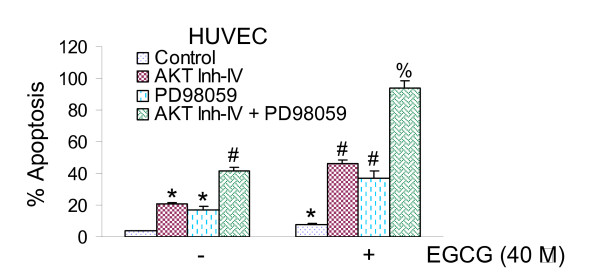
**Interactive effects of AKT inhibitor and MEK1/2 inhibitor on EGCG-induced apoptosis.** Human umbilical vein endothelial cells (HUVECs) were pretreated with AKT inhibitor-IV (1 μM) or MEK1/2 inhibitor PD98059 (10 μM) for 4 h, followed by treatment with EGCG (40 μM) for 2 h. Cells were harvested and TUNEL assay was performed as per manufacturer's instructions (Promega). Data represent mean ± SD. * = significantly different from control, P < 0.05.

We first measured the involvement of PI3K/AKT and MEK/ERK pathways on EGCG-induced apoptosis in HUVEC cells. Treatment of HUVEC cells with AKT inhibitor and MEK1/2 inhibitor (PD98059) alone induced apoptosis (Fig. 1). EGCG had slight but significant effects on apoptosis. The treatment of HUVES cells with the combination of AKT inhibitor and MEK inhibitor induced apoptosis in a synergistic manner. AKT inhibitor and MEK inhibitor, alone or in combination, enhanced the proapoptotic effects of EGCG in HUVEC cells. These data suggest that inhibition of PI3K/AKT and MEK/ERK pathways act synergistically to enhance the antiapoptotic effects of EGCG.

### AKT inhibitor and MEK1/2 inhibitor cooperate to inhibit migration and capillary tube formation of HUVEC cells, and further enhance the inhibitory effects of EGCG on migration and capillary tube formation

**Figure 2:**
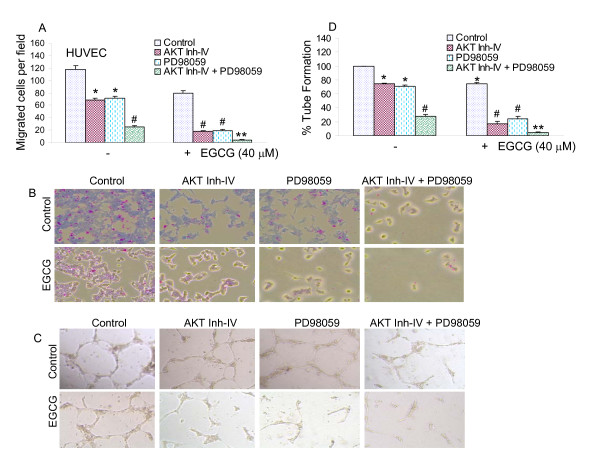
**EGCG inhibits migration and capillary tube formation by HUVEC cells.** (A) Migration of HUVEC cells was assessed using Transwell Boyden chamber containing a polycarbonated filter. HUVECs (4 × 10^4 ^cells) were pretreated with AKT inhibitor IV (1 μM) and/or MEK1/2 inhibitor PD98059 (10 μM) for 2 h, followed by treatment with EGCG (40 μM) or DMSO (control). Migration through the membrane was determined after 24 h of incubation at 37°C. Cells that had migrated to the lower chamber were fixed with 90% methanol, stained with giemsa, quantified by counting the number of cells under a microscope. Data represent mean ± SD. * = significantly different from control, P < 0.05. (B), HUVEC cells were treated as described in A. Cells that had migrated to the lower chamber were fixed with 90% methanol, and photographed with a digital camera attached to a microscope. (C), HUVECs (10 × 10^4^) were seeded in 24-well plates containing matrigel, and pretreated with AKT inhibitor IV (1 μM) and/or MEK1/2 inhibitor PD98059 (10 μM) for 2 h, followed by treatment with EGCG (40 μM) or DMSO (control) for 24 h. Capillary tube structures were photographed with a digital camera attached to a microscope. (D), HUVECs cells were seeded and treated as described in C. Capillary tubes were counted under a microscope. Data represent mean ± SD. * = significantly different from control, P < 0.05.

A critical event in tumor growth and progression is the upregulation of angiogenesis. Thus, targeting angiogenesis has become an attractive treatment modality in cancer medicine. Endothelial cell migration and capillary tube formation are important events for angiogenesis. We first measured the effect of EGCG treatment on migration of HUVEC cells using a modified Boyden Chamber assay (Fig. 2A and 2B). In DMSO-treated controls, a large fraction of HUVEC migrated to the bottom face of the membrane. Treatment of chambers with AKT inhibitor, MEK1/2 inhibitor (PD98059) or EGCG resulted in inhibition of migration of HUVECs. The combination of AKT inhibitor and PD98059 synergistically inhibited cell migration. Interestingly, the inhibitory effects of EGCG on cell migration were further enhanced in the presence of AKT inhibitor and/or MEK1/2 inhibitor (PD98059).

We next examined the effects of PI3K/AKT and MEK/ERK pathways on capillary tube formation by HUVEC on growth factor-reduced matrigel, which is well-accepted technique to measure *in vitro *angiogenesis [57]. The data revealed that AKT inhibitor, MEK1/2 inhibitor (PD98059) and EGCG alone inhibited capillary tube formation by HUVEC cells (Fig. 2C and 2D). The combination of AKT inhibitor and MEK1/2 inhibitor (PD98059) synergistically inhibited capillary tube formation. Interestingly, the inhibitory effects of EGCG on capillary tube formation were further enhanced in the presence of AKT inhibitor and/or MEK1/2 inhibitor (PD98059). These results indicate that the inhibition of PI3K/AKT and MEK/ERK pathways acts synergistically to inhibit migration and capillary tube formation by HUVEC cells.

### FOXO1-TM and FOXO3A-TM (phosphorylation deficient mutants of FOXO) enhance the inhibitory effects of EGCG on migration and capillary tube formation by HUVEC cells

**Figure 3:**
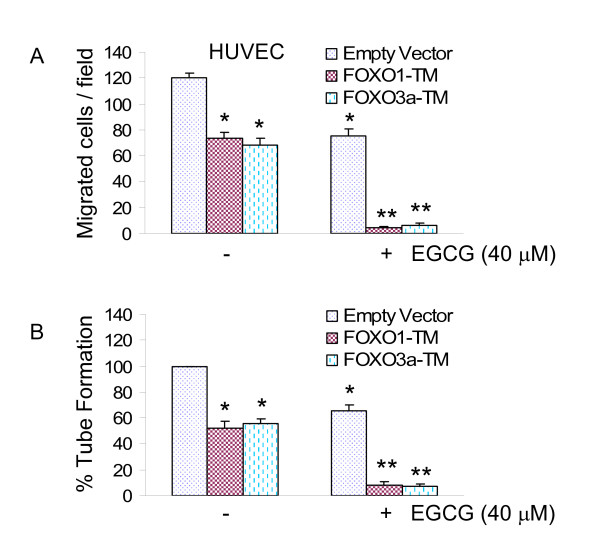
**Phosphorylation deficient mutants of FOXO enhance the inhibitory effects of EGCG on migration and capillary tube formation.** (A) HUVEC (4 × 10^4^) cells were transiently transfected with empty vector, FOXO1-TM or FOXO3A-TM along with pCMV-LacZ vector (as transfection control) and treated with or without EGCG (40 μM). Migration through the membrane was determined after 24 h of incubation at 37°C. Cells that had migrated to the lower chamber were fixed with 90% ethanol, stained with Geimsa, quantified by counting the number of cells under a microscope. Data represent mean ± SD. *, ** = significantly different from control, P < 0.05. (B), HUVEC (4 × 10^4^) cells were transiently transfected with empty vector, FOXO1-TM or FOXO3A-TM along with pCMV-LacZ vector (as transfection control) and treated with or without EGCG (40 μM) for 24 h. Capillary tubes were counted under a microscope. Data represent mean ± SD. *, ** = significantly different from control, P < 0.05.

Recent studies have demonstrated the involvement of FOXO transcription factor in angiogenesis [10,35,36]. We therefore examined the involvement of FOXO transcription factors in EGCG-induced migration and capillary tube formation by HUVEC cells (Fig. 3). Since dephosphorylated FOXO transcription factors translocate to nucleus and induce gene transcription, we use phosphorylation deficient mutants of FOXO (consitutively active) to activate its transcriptional activity. Overexpression of phosphorylation deficient mutants of FOXO (FOXO1-TM and FOXO3a-TM) inhibited migration and capillary tube formation by HUVEC cells. Overexpression of FOXO1-TM and FOXO3a-TM further enhanced the inhibitory effects of EGCG on migration and capillary tube formation by HUVEC cells. These data suggest that FOXO transcription factors may play major role in angiogenesis, and EGCG may inhibit angiogenesis through activation of FOXO.

### AKT inhibitor and MEK1/2 inhibitor synergistically induce FOXO transcriptional activity, and further enhance EGCG-induced FOXO activity in HUVEC cells

**Figure 4:**
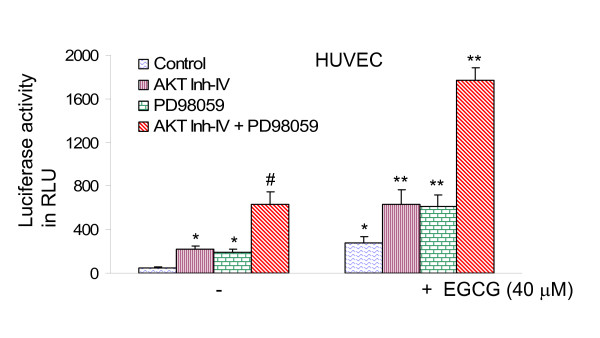
**Inhibition of PI3K/AKT and MEK/ERK pathways synergistically enhanced EGCG-induced FOXO activity in HUVEC cells.** HUVEC cells were transiently transfected with 6X DBE-luciferase and pRL-TK plasmids for 24 h [69]. After transfection, HUVEC cells were pretreated with AKT inhibitor IV (1 μM) and/or MEK1/2 inhibitor PD98059 (10 μM) for 2 h, followed by treatment with or without EGCG (40 μM) for 24 h. Cells were harvested for firefly/Renilla luciferase assays using the Dual-Luciferase Reporter Assay System (Promega). Luciferase counts were normalized using *Renilla *luciferase transfection control. Data represent the mean ± S.D. *, #, ** = significantly different from respective controls, P < 0.05.

We next examined whether inhibition of PI3K/AKT and MEK/ERK pathways act synergistically to induce FOXO transcriptional activity, and inhibition of these two pathways further enhances EGCG-induced FOXO activity (Fig. 4). AKT inhibitor, MEK1/2 inhibitor (PD98059) and EGCG alone induced FOXO transcriptional activity. The combination of AKT inhibitor and PD98059 synergistically induced FOXO activity. Furthermore, the combination of AKT inhibitor and/or PD98059 with EGCG further enhanced FOXO transcriptional activity. These data suggest that inhibition of PI3K/AKT and MEK/ERK pathways act synergistically to induce FOXO transcriptional activity, and inhibition of these two pathways further enhance EGCG-induced FOXO activity.

### Phosphorylation deficient mutants of FOXO induce FOXO transcriptional activity, and further enhance EGCG-induced FOXO activity in HUVEC cells

**Figure 5:**
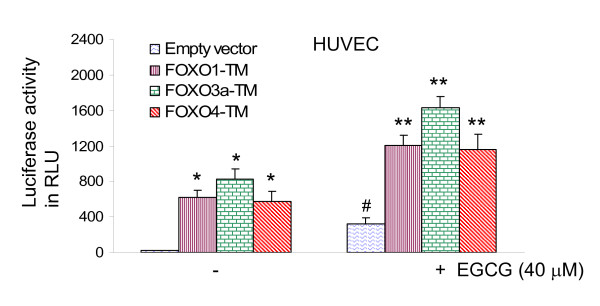
**Phosphorylation deficient mutants of FOXO enhance EGCG-induced FOXO transcriptional activity in HUVEC cells.** HUVEC cells were transiently transfected with empty vector or constructs encoding FOXO1-TM, FOXO3a-TM, or FOXO4-TM together with 6X DBE-luciferase and pRL-TK plasmids for 24 h [69]. Luciferase counts were normalized using *Renilla *luciferase transfection control. After transfection, cells were washed, treated with EGCG (20 μM) for 24 h, and harvested for firefly/Renilla luciferase assays using the Dual-Luciferase Reporter Assay System (Promega). Data represent the mean ± S.D. *, #, ** = significantly different from respective controls, P < 0.05.

We next examined whether EGCG induces transcriptional activation of FOXO in the presence or absence FOXO1-TM, FOXO3a-TM, or FOXO4-TM (phosphorylation deficient triple mutant) (Fig. 5). HUVEC cells were transfected with wild type FOXO promoter linked to a luciferase reporter gene in the presence or absence of plasmids expressing FOXO1-TM, FOXO3a-TM, or FOXO4-TM. After transfection, cells were treated with EGCG for 24 h, and luciferase activity was measured. Transfection of cells with plasmids expressing FOXO1-TM, FOXO3a-TM, or FOXO4-TM induced FOXO transcriptional activity compared with the empty vector (control). EGCG-induced FOXO transcriptional activity was further enhanced in the presence of phosphorylation deficient mutants of FOXO1, FOXO3a, and FOXO4. These data indicate that FOXO transcription factors can mediate the antiangiogenic effects of EGCG.

## Discussion

To the best of our knowledge, this is the first study to demonstrate that inhibition of PI3K/AKT and MEK/ERK pathways acted synergistically to induce activation of FOXO transcription factors and enhanced the antiangiogenic effects of EGCG in HUVEC cells. Specifically, cell migration and capillary tube formation was inhibited by AKT inhibitor and MEK inhibitor. We have recently demonstrated that EGCG can inhibit pancreatic cancer development and angiogenesis in nude mice [57]. EGCG inhibited the expression of genes associated with metastasis and angiogenesis [57]. Our data clearly demonstrate that PI3K/AKT and MEK/ERK pathways converge to regulate angiogenesis through FOXO transcription factor.

Angiogenesis, the sprouting of new capillaries from the preexistent blood vessels, is of central importance in many biological processes, including embryonic vascular development and differentiation, wound healing, and organ regeneration [58,59]. In addition, angiogenesis plays a major role in pathological conditions such as diabetic retinopathy, rheumatoid arthritis, psoriasis, cardiovascular diseases, tumor growth, and metastasis [60,61]. During angiogenesis, endothelial cells migrate, proliferate, organize into tube-like structures, and play an active role in tissue remodeling. This cascade of events is under the control of angiogenic factors which include basic fibroblast growth factor (bFGF) and vascular endothelial growth factor (VEGF) or vascular permeability factor (VPF), an endothelial-cell specific mitogen [58,59]. Both factors have been shown to stimulate the proliferation of vascular endothelial cells and the formation of new capillary beds. In addition to positively acting angiogenic factors, there appears to be several negative regulator of angiogenesis including angiostatin, endostatin, thrombospondin, platelet factor 4, and 16K hPRL [60,62]. Antiangiogenic factors have been shown to antagonize both the proliferation of endothelial cells *in vitro *and neovascularization *in vivo *[60,63]. We have recently shown a positive correlation between inhibition of tumor growth and angiogenesis in mice treated with EGCG. Specifically, EGCG inhibited angiogenesis (vWF, VEGF and CD31) and metastasis (MMP-2, -7, -9 and -12) in nude mice [57]. EGCG also inhibited number of VEGF-R2 positive circulating endothelial cells derived from xenografted nude mice. EGCG inhibited cell migration and capillary tube formation, and these beneficial effects of EGCG were further enhanced in the presence of ERK MAP kinase inhibitor, pointing a positive role of ERK in angiogenesis and metastasis. In the present study, the inhibition of MEK/ERK pathway resulted in inhibition of cell migration and capillary tube formation through activation of FOXO transcription factor.

The Ras signal transduction pathway is complex with multiple intersections and bifurcations [41]. We have recently demonstrated that EGCG can inhibit the expression of Ras and Raf expression in pancreatic cancer cells. Similarly, GTP and EGCG have been shown to inhibit the expression of the K-ras gene, and growth of pancreatic cancer cells [56]. Ras activates three mitogen-activated protein kinases (MAPKs) including ERK, JNK, and p38 [41]. Cells utilize the various Ras-mediated signal transduction pathways to regulate a plethora of phenotypes. Raf-1 contributes directly to ERK activation but not to JNK activation [64]. Since Ras is an upstream target of ERK, the inhibition of Ras/Raf/MEK/ERK pathway by EGCG may be significant to inhibit angiogenesis.

FOXO1 (FKHR) contains 15 consensus phosphorylation sites for the mitogen-activated protein kinase (MAPK) family. The phosphorylation of FOXO1 was demonstrated by ERK and p38 MAPK but not by JNK [51]. Epidermal growth factor or anisomycin increased phosphorylation of exogenous FOXO1, which was significantly inhibited by pretreatment with an MEK 1 inhibitor, PD98059, or a p38 inhibitor, SB203580 [51]. Two-dimensional phosphopeptide mapping using mutation of phosphorylation sites for MAPK revealed that the nine serine residues in FOXO1 are specifically phosphorylated by ERK and that five of the nine residues are phosphorylated by p38 *in vivo *[51]. These data suggest that FOXO1 is specifically phosphorylated by ERK and p38, and that this phosphorylation regulates the function of FOXO1. In the present study, inhibition of Ras/MEK/ERK pathway inhibits capillary tube formation and HUVEC cell migration through activation of FOXO transcription factor.

Activation of AKT through PI3K leads to phosphorylation and nuclear exclusion of the transcription factor FOXO. Accordingly, FOXO1 dephosphorylation leads to translocation to the nucleus and induces the transcription of target genes such as Fas, BIM, p27^/Kip1 ^that trigger apoptosis [16]. With respect to angiogenesis-related molecules, FOXO1 induces the expression of angiopoietin-2 (Ang-2) [3], a competitive antagonist of angiopoietin-1 (Ang-1). Ang-1 and its endothelial receptor tyrosine-protein kinase receptor (Tie2) are required for vascular development, maturation, and stability [65]. Albeit Ang-2 also binds to Tie2, it does not activate this receptor under physiological conditions and is consequently associated with blood vessel destabilization and remodelling [65]. Ang-1 inhibits FOXO1 and thereby the expression of its antagonist Ang-2 [35]. On the opposite, this regulation implies a positive feedback loop in which an increase in Ang-2 expression, blocking Ang-1 effects, leads indirectly to activation of FOXO1 resulting in a further increase in the expression of Ang-2 and other FOXO1 target genes. In the present study, AKT inhibitor blocks capillary tube formation and HUVEC cell migration through transcription activation of FOXO, which may regulate the expression of angiogenesis-related genes. Furthermore, our data demonstrate that inhibition of PI3K/AKT and MEK/ERK pathways converge to inhibit angiogenesis through activation of FOXO transcription factors. Inhibition of these two pathways further enhances the antiangiogenic effects of EGCG.

## Conclusion

We have demonstrated that inhibition of PI3K/AKT and Ras/MEK/ERK pathways interact synergistically to activate FOXO transcription factors which, in turn, inhibit angiogenesis. Inhibition of both of these pathways further enhances the antiangiogenic effects of EGCG. In a recent study, we have demonstrated that EGCG can modulate the expression of genes known to play a role in the cancer progression, invasion, metastasis and angiogenesis [57]. We have also demonstrated that EGCG can induce apoptosis through multiple mechanisms i.e. activation of caspases, regulation of Bcl-2 family members, generation of ROS, inhibition of Raf-1, ERK and upregulation of JNK and p38 MAP kinase pathways [66]. The activation of FOXO transcription factors through inhibition of Ras/MEK/ERK and PI3K/AKT pathways may have physiological significance in management of diabetic retinopathy, rheumatoid arthritis, psoriasis, cardiovascular diseases, and cancer.

## Methods

### Reagents

EGCG was purchased from LKT Laboratories, Inc. (St. Paul, MN). MEK inhibitor (PD98059), AKT inhibitor-IV and Terminal Deoxynucleotidyl Transferase Biotin-dUTP Nick End Labeling (TUNEL) assay kit were purchased from EMD Biosciences (San Diego, CA). AKT inhibitor-IV is a cell-permeable benzimidazole compound that inhibits AKT phosphorylation/activation by targeting the ATP binding site of a kinase upstream of AKT, but downstream of PI3K [67]. It has been shown to block AKT-mediated FOXO1a nuclear export and cell proliferation [67]. Unlike phosphatidylinositol analog-based AKT inhibitors, this inhibitor does not affect PI3K [67]. Dual Luciferase Reporter Assay kit was purchased from Promega Corporation (Madison, WI).

### Cell Culture and cell survival assay

Human umbilical vein endothelial cells (HUVECs) were purchased from Clonetics (Walkersville, MD) and maintained in endothelial cell growth factor medium-2 (EGM2 MV SingleQuots, Clonetics) supplemented with 5% FBS. Stock solutions of the EGCG were prepared in DMSO and diluted with complete medium, and an equal volume of DMSO (final concentration, 0.05%) was added to the controls.

### Capillary tube formation assay

Cell migration assay was performed as we described earlier [68]. In brief, matrigel (100 μl) was added to wells of a 96-well culture plate and allowed to polymerize for 1 h at 37°C. To examine the effects of EGCG on *in vitro *angiogenesis, subconfluent HUVECs were resuspended in complete medium and added to Matrigel containing wells (1 × 10^4 ^cells/well), and exposed to EGCG or DMSO (control). The plates were incubated at 37°C in a humidified atmosphere of 95% air and 5% CO_2_. Capillary tube formation was assessed after 24 h by counting the total number of capillary like tubular structures from three randomly chosen fields using an inverted microscope.

### *In vitro *cell migration assay

Cell migration assay was performed as we described earlier [68]. In brief, migration of HUVEC cells was assessed using Transwell Boyden chamber (Corning, Acton, MA) containing a polycarbonated filter with a pore size of 8-μM. HUVECs (4 × 10^4 ^cells in 0.2 ml) cells in complete medium was mixed with desired concentration of EGCG or DMSO (control), and the cell suspension was added to the upper chamber. The lower chamber contained 0.6 ml of complete medium with the same concentration of EGCG or DMSO. Migration through the membrane was determined after 24 h of incubation at 37°C. Cells remaining on the topside of the transwell membrane were removed using a cotton swab. The membrane was washed with ice-cold PBS. Cells that had migrated to the underside were fixed with 90% methanol and stained with giemsa. Cell migration was quantified by counting the number of cells per field in five random fields.

### TUNEL assay

HUVECs cells were pretreated with AKT inhibitor-IV (1 μM) or MEK1/2 inhibitor PD98059 (10 μM) in the presence or absence of EGCG (40 μM). Cells were harvested and TUNEL assay was performed as per manufacturer's instructions (Promega).

### Luciferase assay

HUVEC cells were transfected with empty vector, FOXO1-TM, FOXO3a-TM or FOXO4-TM along with reporter plasmids, p6xDBE-luc and pRL-TK. The FOXO expression vectors (wild type and phosphorylation deficient mutants) and FOXO-luciferase constructs have been described elsewhere [10,69]. After 24 h, transfection medium was replaced with culture medium and cells were treated with EGCG (40 μM). After incubation of 24 h, the relative luciferase activity, i.e. firefly enzyme activity divided by that of the Renilla enzyme, was determined using Dual Luciferase Reporter Assay System (Promega) according to the manufacturer's protocol.

## Statistical analysis

The mean and SD were calculated for each experimental group. Differences between groups were analyzed by one or two way ANOVA using PRISM statistical analysis software (GrafPad Software, Inc., San Diego, CA). Significant differences among groups were calculated at P < 0.05.

## Competing interests

The author(s) declare that they have no competing interests.

## References

[B1] Folkman J (2002). Role of angiogenesis in tumor growth and metastasis. Semin Oncol.

[B2] Folkman J (2004). A novel anti-vascular therapy for cancer. Cancer Biol Ther.

[B3] Daly C, Wong V, Burova E, Wei Y, Zabski S, Griffiths J, Lai KM, Lin HC, Ioffe E, Yancopoulos GD, Rudge JS (2004). Angiopoietin-1 modulates endothelial cell function and gene expression via the transcription factor FKHR (FOXO1). Genes Dev.

[B4] Kitamura T, Nakae J, Kitamura Y, Kido Y, Biggs WH, Wright CV, White MF, Arden KC, Accili D (2002). The forkhead transcription factor Foxo1 links insulin signaling to Pdx1 regulation of pancreatic beta cell growth. J Clin Invest.

[B5] Castrillon DH, Miao L, Kollipara R, Horner JW, DePinho RA (2003). Suppression of ovarian follicle activation in mice by the transcription factor Foxo3a. Science.

[B6] Nakae J, Biggs WH, Kitamura T, Cavenee WK, Wright CV, Arden KC, Accili D (2002). Regulation of insulin action and pancreatic beta-cell function by mutated alleles of the gene encoding forkhead transcription factor Foxo1. Nat Genet.

[B7] Xia SJ, Pressey JG, Barr FG (2002). Molecular pathogenesis of rhabdomyosarcoma. Cancer Biol Ther.

[B8] Hu MC, Lee DF, Xia W, Golfman LS, Ou-Yang F, Yang JY, Zou Y, Bao S, Hanada N, Saso H (2004). IkappaB kinase promotes tumorigenesis through inhibition of forkhead FOXO3a. Cell.

[B9] Hosaka T, Biggs WH, Tieu D, Boyer AD, Varki NM, Cavenee WK, Arden KC (2004). Disruption of forkhead transcription factor (FOXO) family members in mice reveals their functional diversification. Proc Natl Acad Sci USA.

[B10] Furuyama T, Kitayama K, Shimoda Y, Ogawa M, Sone K, Yoshida-Araki K, Hisatsune H, Nishikawa S, Nakayama K, Nakayama K, Ikeda K, Motoyama N, Mori N (2004). Abnormal angiogenesis in Foxo1 (Fkhr)-deficient mice. J Biol Chem.

[B11] Galili N, Davis RJ, Fredericks WJ, Mukhopadhyay S, Rauscher FJ, Emanuel BS, Rovera G, Barr FG (1993). Fusion of a fork head domain gene to PAX3 in the solid tumour alveolar rhabdomyosarcoma. Nat Genet.

[B12] Anderson MJ, Viars CS, Czekay S, Cavenee WK, Arden KC (1998). Cloning and characterization of three human forkhead genes that comprise an FKHR-like gene subfamily. Genomics.

[B13] Hillion J, Le Coniat M, Jonveaux P, Berger R, Bernard OA (1997). AF6q21, a novel partner of the MLL gene in t(6;11)(q21;q23), defines a forkhead transcriptional factor subfamily. Blood.

[B14] Borkhardt A, Repp R, Haas OA, Leis T, Harbott J, Kreuder J, Hammermann J, Henn T, Lampert F (1997). Cloning and characterization of AFX, the gene that fuses to MLL in acute leukemias with a t(X;11)(q13;q23). Oncogene.

[B15] Van Der Heide LP, Hoekman MF, Smidt MP (2004). The ins and outs of FoxO shuttling: mechanisms of FoxO translocation and transcriptional regulation. Biochem J.

[B16] Brunet A, Bonni A, Zigmond MJ, Lin MZ, Juo P, Hu LS, Anderson MJ, Arden KC, Blenis J, Greenberg ME (1999). Akt promotes cell survival by phosphorylating and inhibiting a Forkhead transcription factor. Cell.

[B17] Guo S, Rena G, Cichy S, He X, Cohen P, Unterman T (1999). Phosphorylation of serine 256 by protein kinase B disrupts transactivation by FKHR and mediates effects of insulin on insulin-like growth factor-binding protein-1 promoter activity through a conserved insulin response sequence. J Biol Chem.

[B18] Medema RH, Kops GJ, Bos JL, Burgering BM (2000). AFX-like Forkhead transcription factors mediate cell-cycle regulation by Ras and PKB through p27kip1. Nature.

[B19] Nakamura N, Ramaswamy S, Vazquez F, Signoretti S, Loda M, Sellers WR (2000). Forkhead transcription factors are critical effectors of cell death and cell cycle arrest downstream of PTEN. Mol Cell Biol.

[B20] Dijkers PF, Medema RH, Pals C, Banerji L, Thomas NS, Lam EW, Burgering BM, Raaijmakers JA, Lammers JW, Koenderman L, Coffer PJ (2000). Forkhead transcription factor FKHR-L1 modulates cytokine-dependent transcriptional regulation of p27(KIP1). Mol Cell Biol.

[B21] Dijkers PF, Birkenkamp KU, Lam EW, Thomas NS, Lammers JW, Koenderman L, Coffer PJ (2002). FKHR-L1 can act as a critical effector of cell death induced by cytokine withdrawal: protein kinase B-enhanced cell survival through maintenance of mitochondrial integrity. J Cell Biol.

[B22] Cappellini A, Tabellini G, Zweyer M, Bortul R, Tazzari PL, Billi AM, Fala F, Cocco L, Martelli AM (2003). The phosphoinositide 3-kinase/Akt pathway regulates cell cycle progression of HL60 human leukemia cells through cytoplasmic relocalization of the cyclin-dependent kinase inhibitor p27(Kip1) and control of cyclin D1 expression. Leukemia.

[B23] Burgering BM, Kops GJ (2002). Cell cycle and death control: long live Forkheads. Trends Biochem Sci.

[B24] Dijkers PF, Medema RH, Lammers JW, Koenderman L, Coffer PJ (2000). Expression of the pro-apoptotic Bcl-2 family member Bim is regulated by the forkhead transcription factor FKHR-L1. Curr Biol.

[B25] Gilley J, Coffer PJ, Ham J (2003). FOXO transcription factors directly activate bim gene expression and promote apoptosis in sympathetic neurons. J Cell Biol.

[B26] Tang TT, Dowbenko D, Jackson A, Toney L, Lewin DA, Dent AL, Lasky LA (2002). The forkhead transcription factor AFX activates apoptosis by induction of the BCL-6 transcriptional repressor. J Biol Chem.

[B27] Perugini RA, McDade TP, Vittimberga FJ, Callery MP (2000). Pancreatic cancer cell proliferation is phosphatidylinositol 3-kinase dependent. J Surg Res.

[B28] Shah SA, Potter MW, Hedeshian MH, Kim RD, Chari RS, Callery MP (2001). PI-3' kinase and NF-kappaB cross-signaling in human pancreatic cancer cells. J Gastrointest Surg.

[B29] Bondar VM, Sweeney-Gotsch B, Andreeff M, Mills GB, McConkey DJ (2002). Inhibition of the phosphatidylinositol 3'-kinase-AKT pathway induces apoptosis in pancreatic carcinoma cells in vitro and in vivo. Mol Cancer Ther.

[B30] Cheng JQ, Ruggeri B, Klein WM, Sonoda G, Altomare DA, Watson DK, Testa JR (1996). Amplification of AKT2 in human pancreatic cells and inhibition of AKT2 expression and tumorigenicity by antisense RNA. Proc Natl Acad Sci USA.

[B31] Ruggeri BA, Huang L, Wood M, Cheng JQ, Testa JR (1998). Amplification and overexpression of the AKT2 oncogene in a subset of human pancreatic ductal adenocarcinomas. Mol Carcinog.

[B32] Altomare DA, Tanno S, De Rienzo A, Klein-Szanto AJ, Tanno S, Skele KL, Hoffman JP, Testa JR (2003). Frequent activation of AKT2 kinase in human pancreatic carcinomas. J Cell Biochem.

[B33] Schlieman MG, Fahy BN, Ramsamooj R, Beckett L, Bold RJ (2003). Incidence, mechanism and prognostic value of activated AKT in pancreas cancer. Br J Cancer.

[B34] Dejana E, Taddei A, Randi AM (2007). Foxs and Ets in the transcriptional regulation of endothelial cell differentiation and angiogenesis. Biochim Biophys Acta.

[B35] Chlench S, Mecha Disassa N, Hohberg M, Hoffmann C, Pohlkamp T, Beyer G, Bongrazio M, Da Silva-Azevedo L, Baum O, Pries AR, Zakrzewicz A (2007). Regulation of Foxo-1 and the angiopoietin-2/Tie2 system by shear stress. FEBS Lett.

[B36] Potente M, Urbich C, Sasaki K, Hofmann WK, Heeschen C, Aicher A, Kollipara R, DePinho RA, Zeiher AM, Dimmeler S (2005). Involvement of Foxo transcription factors in angiogenesis and postnatal neovascularization. J Clin Invest.

[B37] Boguski MS, McCormick F (1993). Proteins regulating Ras and its relatives. Nature.

[B38] Bokoch GM, Der CJ (1993). Emerging concepts in the Ras superfamily of GTP-binding proteins. Faseb J.

[B39] Gibbs JB, Sigal IS, Poe M, Scolnick EM (1984). Intrinsic GTPase activity distinguishes normal and oncogenic ras p21 molecules. Proc Natl Acad Sci USA.

[B40] Shih C, Weinberg RA (1982). Isolation of a transforming sequence from a human bladder carcinoma cell line. Cell.

[B41] Campbell SL, Khosravi-Far R, Rossman KL, Clark GJ, Der CJ (1998). Increasing complexity of Ras signaling. Oncogene.

[B42] Schaeffer HJ, Weber MJ (1999). Mitogen-activated protein kinases: specific messages from ubiquitous messengers. Mol Cell Biol.

[B43] Han J, Ulevitch RJ (1999). Emerging targets for anti-inflammatory therapy. Nat Cell Biol.

[B44] Davis RJ (2000). Signal transduction by the JNK group of MAP kinases. Cell.

[B45] Chang L, Karin M (2001). Mammalian MAP kinase signalling cascades. Nature.

[B46] Robinson MJ, Cobb MH (1997). Mitogen-activated protein kinase pathways. Curr Opin Cell Biol.

[B47] Woessmann W, Meng YH, Mivechi NF (1999). An essential role for mitogen-activated protein kinases, ERKs, in preventing heat-induced cell death. J Cell Biochem.

[B48] Kyriakis JM, Avruch J (1996). Sounding the alarm: protein kinase cascades activated by stress and inflammation. J Biol Chem.

[B49] Ono K, Han J (2000). The p38 signal transduction pathway: activation and function. Cell Signal.

[B50] Lubinus M, Meier KE, Smith EA, Gause KC, LeRoy EC, Trojanowska M (1994). Independent effects of platelet-derived growth factor isoforms on mitogen-activated protein kinase activation and mitogenesis in human dermal fibroblasts. J Biol Chem.

[B51] Asada S, Daitoku H, Matsuzaki H, Saito T, Sudo T, Mukai H, Iwashita S, Kako K, Kishi T, Kasuya Y, Fukamizu A (2007). Mitogen-activated protein kinases, Erk and p38, phosphorylate and regulate Foxo1. Cell Signal.

[B52] Manson MM, Farmer PB, Gescher A, Steward WP (2005). Innovative agents in cancer prevention. Recent Results Cancer Res.

[B53] Shankar S, Ganapathy S, Srivastava RK (2007). Green tea polyphenols: biology and therapeutic implications in cancer. Front Biosci.

[B54] Park OJ, Surh YJ (2004). Chemopreventive potential of epigallocatechin gallate and genistein: evidence from epidemiological and laboratory studies. Toxicol Lett.

[B55] Lambert JD, Yang CS (2003). Mechanisms of cancer prevention by tea constituents. J Nutr.

[B56] Lyn-Cook BD, Rogers T, Yan Y, Blann EB, Kadlubar FF, Hammons GJ (1999). Chemopreventive effects of tea extracts and various components on human pancreatic and prostate tumor cells in vitro. Nutr Cancer.

[B57] Shankar S, Ganapathy S, Hingorani SR, Srivastava RK (2008). EGCG inhibits growth, invasion, angiogenesis and metastasis of pancreatic cancer. Front Biosci.

[B58] Folkman J (2003). Fundamental concepts of the angiogenic process. Curr Mol Med.

[B59] Folkman J (2003). Angiogenesis and proteins of the hemostatic system. J Thromb Haemost.

[B60] Folkman J (2003). Angiogenesis inhibitors: a new class of drugs. Cancer Biol Ther.

[B61] Folkman J (2003). Angiogenesis and apoptosis. Semin Cancer Biol.

[B62] Nyberg P, Xie L, Kalluri R (2005). Endogenous inhibitors of angiogenesis. Cancer Res.

[B63] Folkman J (2004). Endogenous angiogenesis inhibitors. Apmis.

[B64] Minden A, Lin A, McMahon M, Lange-Carter C, Derijard B, Davis RJ, Johnson GL, Karin M (1994). Differential activation of ERK and JNK mitogen-activated protein kinases by Raf-1 and MEKK. Science.

[B65] Maisonpierre PC, Suri C, Jones PF, Bartunkova S, Wiegand SJ, Radziejewski C, Compton D, McClain J, Aldrich TH, Papadopoulos N (1997). Angiopoietin-2, a natural antagonist for Tie2 that disrupts in vivo angiogenesis. Science.

[B66] Shankar S, Suthakar G, Srivastava RK (2007). Epigallocatechin-3-gallate inhibits cell cycle and induces apoptosis in pancreatic cancer. Front Biosci.

[B67] Kau TR, Schroeder F, Ramaswamy S, Wojciechowski CL, Zhao JJ, Roberts TM, Clardy J, Sellers WR, Silver PA (2003). A chemical genetic screen identifies inhibitors of regulated nuclear export of a Forkhead transcription factor in PTEN-deficient tumor cells. Cancer Cell.

[B68] Shankar S, Chen Q, Sarva K, Siddiqui I, Srivastava RK (2007). Curcumin enhances the apoptosis-inducing potential of TRAIL in prostate cancer cells: molecular mechanisms of apoptosis, migration and angiogenesis. J Mol Signal.

[B69] Furukawa-Hibi Y, Kobayashi Y, Chen C, Motoyama N (2005). FOXO transcription factors in cell-cycle regulation and the response to oxidative stress. Antioxid Redox Signal.

